# 

**Published:** 1884-03

**Authors:** 


MARCH 1884.

### THE AMERICAN JOURNAL OF [illegible]ENTAL SCIENCE.

PUBLISHED MONTHLY.

$2.50 PER ANNUM.

EDITED BY

F. J. S. GORGAS, M. D., D. D. S.

Vol. 17. THIRD SERIES. No. 11.

BALTIMORE:

SNOWDEN & COWMAN.

LONDON:
TRUBNER & CO., 60 PATERNOSTER ROW.

1883.

PAYABLE IN ADVANCE.

